# Challenges in Clinical Diagnosis and Management of Chronic Endometritis

**DOI:** 10.3390/diagnostics12112711

**Published:** 2022-11-05

**Authors:** Tadahiro Yasuo, Kotaro Kitaya

**Affiliations:** 1 Department of Obstetrics and Gynecology, Otsu City Hospital, Otsu 520-0804, Japan; 2 Infertility Center, Kouseikai Mihara Hospital/Katsura Mihara Clinic, 6–8 Kamikatsura Miyanogo-cho, Nishikyo-ku, Kyoto 615-8227, Japan

**Keywords:** CD138, chronic endometritis, histopathology, hysteroscopy, microbiome analysis

## Abstract

Chronic endometritis (CE) is a local mucosal infectious and inflammatory disorder characterized by unusual filtration of CD138(+) endometrial stromal plasmacytes. CE is attracting attention due to its potential association with infertility of unknown etiology, repeated implantation failure, recurrent pregnancy loss, and several maternal/neonatal complications. Due to the variance in study design among researchers, universal diagnostic criteria remain to be established for the clinical diagnosis and management of CE. This review article aims to summarize current knowledge and provide insights into unsolved questions on CE to establish clinical guidelines for the disease from the viewpoint of human reproduction.

## 1. Introduction

The human endometrium is a unique mucosal tissue inside the uterine corpus that repeats dynamic morphologic, transcriptional, and translational changes in a monthly cycle under the orchestration of the two major ovarian steroids, 17beta-estradiol and progesterone [1]. One monthly cycle (menstrual cycle) is composed of multiple biological events such as menstruation (mucosal shedding), proliferation (of epithelial, stromal, and vascular cells), secretion (of mucus and soluble molecules), and decidualization (morphological and functional changes for the establishment of conception). The menstrual cycle is suspended when one or more embryos successfully implant, or ovarian steroidogenesis is lost due to aging, anti-cancer chemotherapy, irradiation, or other causes [2]. During the menstrual cycle, the human endometrium is infiltrated by a wide variety of immunocompetent cells, such as natural killer (NK) cells, macrophages, neutrophils, dendritic cells, and subsets of T cells. The composition and density of these endometrial leukocyte subpopulations also fluctuate periodically within a menstrual cycle. Such timely transitions in mucosal leukocyte subpopulations are considered to play a critical role in the establishment of embryo implantation and placentation [3].

By contrast, antibody-bearing lymphocytes of the B cell lineage are a rare leukocyte subpopulation found in the nonpathological human endometrium. Under a physiological condition, endometrial B cells account for approximately 3% of all endometrial lymphocytes and no more than 2% of all endometrial cells throughout the menstrual cycle [4]. Some CD138(+) endometrial stromal plasmacytes (ESPCs) can occasionally be seen in the stromal compartments. Endometrial B cells exhibit a rather naïve phenotype, with a higher expression level of typical B cell-associated genes, such as CD19, MS4A1 (encoding CD20), CD79A, LY9, CD83, BTG1, and CXCR4, and a moderate level of CD74 and HLA-DRA, compared with endometrial NK cells, macrophages, and dendritic cells (the data are known to the peer-reviewers of the manuscript but were not retrieved for the article [5]). Some CD22(+) B cells are also observed in the center of the lymphoid aggregate, a unique structure that is present in the stromal compartments of the endometrial stratum basalis, surrounded by several hundred leukocytes, including T cells, monocytes/macrophages, and NK cells. Additionally, various immunoglobulins (Ig), including heavy chains of IgG, IgM, IgJ, IgA1, and IgA2, and light chains of Igκ, are also detectable in the nonpathological human endometrium, suggesting that these Igs are, at least in part, considered to be locally produced by endometrial B cells and/or ESPCs [6].

Chronic endometritis (CE) is a localized infectious/inflammatory disorder of the uterine mucosal lining [7,8]. In striking contrast to acute endometritis characterized by systemic fever, pelvic pain, and increased vaginal discharge, such subjective symptoms and objective findings of CE are so subtle and nondescript that it is often overlooked by affected patients and experienced gynecologists [6]. The asymptomatic or oligosymptomatic nature of CE has hindered basic and clinical research on this disease. There are some controversies over the use of the term “chronic” in the description of this pathology, given that the onset, progress, and remission of CE remain fully determined. Recent studies, however, have demonstrated that CE is frequently found in infertile women with a history of unknown etiology, endometriosis, repeated implantation failure (RIF) following in vitro fertilization–embryo transfer (IVF-ET) cycles, and recurrent pregnancy loss [9,10,11,12]. In addition, chronic deciduitis, a persistent form of CE during pregnancy, is reported to be associated with some obstetric/neonatal complications, such as preterm labor, pre-eclampsia, periventricular leukomalacia, and cerebral palsy in premature infants [6,12,13,14,15,16]. Meanwhile, little is known about the relationship between CE and gynecologic malignant diseases.

The histopathologic features of CE are superficial mucosal edema, increased endometrial stromal cell density, unsynchronized differentiation between endometrial epithelial cells (EECs) and stromal cells, and unusual invasions by ESPCs [6,7,8]. Of these histopathologic features, the most specific and sensitive findings of CE are thought to be the presence of multiple ESPCs. ESPCs are observed in the endometrial stromal compartments as scattered cells or clustered cells. Oral antibiotic agents have been prescribed and found to be effective in the histopathologic eradication of ESPCs in affected infertile women [17,18,19,20,21,22,23]. However, whether the cure for histopathologic CE improves the reproductive outcomes of subsequent infertility treatment cycles in these women remains fully unknown as the definitive diagnostic criteria and/or universal guidelines for CE are lacking. Moreover, several problems arise in the clinical management of CE in infertile women. In this review article, we aimed to summarize current knowledge on CE and provide insights into the unresolved problems regarding its clinical diagnosis and management.

## 2. Challenges in Clinical Diagnosis of Histopathologic CE

### 2.1. How Can We Accurately Identify ESPCs in Histopathologic Evaluation?

**Figure 1:**
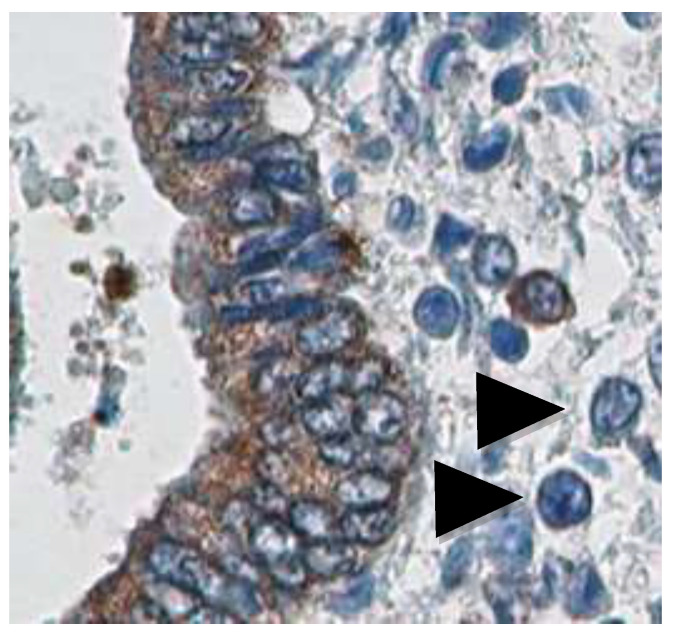
Effectiveness of IHC-CD138 for identification of ESPCs. Light microscopic image of human endometrium with IHC-CD138 and hematoxylin nuclear staining. The tissue contains several cells with spoke-wheel/clock-face patterns (arrows) but is negative for CD138, which is difficult to distinguish via conventional tissue staining alone. Reprinted from Kitaya et al., Current understanding of chronic endometritis. Diagnostic. Histopathol. 2013, 19, 231–237 (Copyright Clearance Center Number 5407080987200, RightsLink/Elsevier).

Histopathologic examinations have been traditionally utilized in the diagnosis of CE, but the identification of ESPCs alone via the conventional tissue staining method is challenging and demanding for clinical pathologists. Under light microscopy, blood plasmacytes typically appear as large lymphocytes with a high nucleus/cytoplasm ratio, basophilic cytoplasm, and eccentric nuclei with heterochromatin rearrangement called the “spoke-wheel” or “clock-face” pattern. However, several endometrium-component cell types (such as NK cells, macrophages, and stromal fibroblasts) present a morphological appearance that resembles ESPCs [6] (Figure 1, Table 1). CD138 (also known as syndecan-1) is a heparan sulfate/chondroitin sulfate proteoglycan expressed on the plasma membrane of plasmacytes. The introduction of immunohistochemistry for CD138 (IHC-CD138) markedly (odds ratio: 2.8) improved the sensitivity (100% vs. 75%), specificity (100% vs. 65%), interobserver variability (96% vs. 68%), and intraobserver variability (93% vs. 47%) in the histopathologic diagnosis of CE [24,25].

Regardless of the development and spread of IHC-CD138 in the diagnosis of CE, there are several problems in its clinical utilization. First, the cells that express CD138 in the human endometrium are not only ESPCs. EECs also constitutively express CD138 on the plasma membrane, particularly on their basolateral sides. The primary monoclonal antibodies against CD138 on ESPCs also react to the epitope of CD138 on EECs, although the immunoreactivity in EECs is relatively weaker compared with ESPCs [6]. The conditions of the section preparation and staining intensity may cause a mix-up between ESPCs and EECs, potentially leading to the overdiagnosis of CE. The combination of IHC-CD138 and conventional nucleic staining may minimize this type of misinterpretation of the findings. Another promising marker for ESPCs is multiple myeloma oncogene (MUM)-1, a transcription factor expressed in the late plasma cell-directed stages of differentiating B cells. A recent study showed that sensitivity and specificity in the detection of ESPCs were higher in IHC-CD138 than in immunohistochemistry for MUM-1, but MUM-1 scored higher in inter-observer agreement compared to CD138 [26]. The combination of IHC-CD138 and MUM-1 may potentially make up for the shortcomings of each molecular marker in the detection of ESPCs.

Second, standardized techniques and conditions are not yet established for IHC-CD138 for human endometrium. Thus, the diagnosis of CE is potentially affected by multiple laboratory factors such as the types of primary antibodies, the conditions for secondary detection systems, and the thickness, area, and number of fields and/or sections observed in the detection of ESPCs. Indeed, studies reported that dilution of the primary antibody had an impact on the histopathologic diagnostic rates of CE in infertile women [27,28]. An optimal setting in laboratory examinations is essential for the accurate evaluation of ESPCs. Nordic immunohistochemical Quality Control (NordiQC) is a professional and scientific organization of pathologists who promote the quality of immunohistochemistry and expand its clinical use. On their website (https://www.nordiqc.org/epitope.php?id=37 (accessed on 4 November 2022)), NordiQC provides the assessment results and recommended protocols of IHC-CD138. Its contents may help standardize the techniques and conditions regarding IHC-CD138 for the human endometrium [29].

Third, the sampling method and device used in endometrial biopsy are other important factors that can affect the diagnostic performance of CE. ESPCs accumulate focally in the endometrial stromal compartments rather than distributing evenly; thus, ESPCs may be missed in small endometrial biopsy specimens [30]. In some women with CE, ESPCs amass only in the endometrial basal layer [31]. Examinations using the tissues obtained via “whole-wall” endometrial curettage may raise the possibility of detecting ESPCs, but this sampling method can be harmful to women desiring pregnancy, causing complications such as endometrial thinning and intrauterine adhesions/Asherman’s syndrome, which deteriorate their reproductive outcomes. Thus, the best strategy for endometrial biopsy remains uncertain. Moreover, many studies agree that the detection rate of ESPCs is higher in the proliferative phase than in the secretory phase [32,33,34]. A recent study also demonstrated that the ESPC density is higher in the proliferative phase than in the secretory phase, regardless of whether ESPCs are scattered (25.3% versus 1.5%) or clustered (13.2% versus 0%) in women with CE. Interestingly, the likelihood of detecting ESPCs is higher in the early proliferative phase (on days 5–8 of the menstrual cycle) that in the late proliferative phase (on days 9–14) [35]. Thus, the interpretation of the IHC-CD138 results should be detailed and optimized according to the date on which the endometrial biopsy was performed in the menstrual cycle.

### 2.2. What Is the Optimal ESPC Density Cutoff Value or Threshold for Infertile Women with CE?

The cutoff value/threshold for ESPC density has not yet been defined for the histopathologic diagnosis of CE, particularly for infertile women. While experts consider that the presence of multiple ESPCs is a prerequisite for the diagnosis of CE, a few CD138(+) ESPCs are reported to be found in 30% of healthy fertile women [36].

Song et al. [37] compared the effectiveness of medical intervention (14-day oral administration of the antibiotic agents levofloxacin (500 mg/day) and tinidazole (1000 mg/day), n = 59) vs. expectant management (no treatment, with the hope that the patient will be cured following menstrual shedding of the endometrium, n = 55) to treat histopathologic CE (defined as ≥1 CD138(+) ESPC in 10 light microscopic high-power fields (HPFs)) in a single-blind randomized controlled trial. The cure rate of histopathologic CE was much higher in the antibiotic treatment group (89.3%) than in the no-treatment group (12.7%), confirming the results of the previous case–control study, which found that natural remission of CE does not happen often following menstruation [22]. When mild CE was defined as <10 CD138(+) ESPCs in 10 HPFs, and severe CE as ≥10 CD138(+) ESPCs in 10 HPFs, the cure rate of the histopathologic CE (<1 CD138(+) ESPC in 10 HPFs) in the antibiotic treatment group was significantly lower in women with severe CE than in those with mild CE (97.7% vs. 68.8%, *p* < 0.001). The results went for the no-treatment group (17.5% in mild CE vs. 0% in severe CE, *p* < 0.001), implying that 10 CD138(+) ESPCs in 10 HPFs may be a potential cutoff value/threshold for ESPC density for determining the severity and intractability of histopathologic CE.

Meanwhile, from the point of view of reproductive outcomes, the optimal cutoff value/threshold for ESPC density in CE remains controversial. In a prospective observational study, Hirata et al. [38] recruited 53 infertile women undergoing single vitrified–warmed blastocyst transfer in a hormone replacement cycle. Four histopathologic criteria (≥1, ≥2, ≥3, and ≥5 ESPCs in 10 HPFs) were set to define CE that potentially affected the reproductive outcomes of these women. The authors found that when histopathologic CE was defined as ≥1 CD138(+) ESPCs in 10 HPFs, the reproductive outcome was poorer in the CE group than in the non-CE group (clinical pregnancy rate (CPR): 30.8% vs. 63.0%, live birth rate (LBR): 7.7% vs. 51.9%, and miscarriage rate (MR): 75% vs. 17.7%) with a sensitivity of 87.5% and specificity of 64.9%.

By contrast, in another prospective study that enrolled 80 infertile women undergoing their first vitrified–warmed euploid blastocyst transfer cycles, Herlihy et al. [39] demonstrated that 49 percent of endometrial samples had ≥1 ESPC, 11% had ≥5 ESPCs, and 4% had ≥10 ESPCs in 10 HPFs. When CE was defined as 1 ESPC in 10 HPFs, the LBR in the euploid blastocyst transfer cycles was at a similar level (*p* = 0.18) between the CE group (56%) and the non-CE group (42%), suggesting overdiagnosis and overtreatment of CE according to this diagnostic criterion. The results were the same (*p* = 0.33, 33% in the CE group and 51% in the non-CE group) when CE was defined as 5 ESPCs in 10 HPFs. These findings suggest that CE with low ESPC density may not impair endometrial receptivity in women undergoing vitrified–warmed euploid blastocyst transfer cycles; however, the study was underpowered in its ability to detect a significant difference, and it was concluded that a larger study is required to answer the question.

Meta-analysis is an important tool to discuss the cutoff value/threshold for ESPC density for women seeking to become pregnant, but it is very difficult to draw conclusions from a small sample size used in a single study. The report by Cheng et al. [20] included nine studies that defined CE as 1 ESPC in all of the endometrial sections observed in women with a history of RIF. The reproductive outcome measures, including implantation rate, ongoing pregnancy rate (OPR)/LBR, and CPR, were worse in RIF women with persistent CE following antibiotic treatment than in those with cured CE, whereas MR was at a similar level between the two groups. Antibiotic treatment may therefore improve the reproductive outcomes of RIF women in subsequent ET cycles only when the cure of CE is confirmed histopathologically, although the cutoff values/thresholds for ESPC density varied among the studies.

In a meta-analysis of eight studies, Vitagliano et al. [40] demonstrated a lower OPR/LBR (OR = 1.97, 95% CI = 1.11–3.48, *p* = 0.02) and CPR (OR = 2.28, 95% CI = 1.34–3.86, *p* = 0.002) in infertile women with CE than in those without CE (defined as ≥1 CD138(+)-ESPC in 1 HPF = 10 ESPCs in 10 HPFs), whereas there was no difference in the MR. Additionally, a subgroup analysis of two studies disclosed that severe CE (defined as ≥50 ESPCs in 10 HPFs) was associated with poor reproductive outcomes (OPR/LBR: OR = 0.43, 95% CI = 0.25–0.74, *p* = 0.003, and CPR: OR = 0.40, 95% CI = 0.24–0.68, *p* = 0.0007) compared to mild CE (defined as 10–40 CD138(+) ESPCs in 10 HPFs), but again, without any difference in the MR. By contrast, there was no difference between mild CE and non-CE in terms of the OPR/LBR, CPR, and MR.

Collectively, the current evidence is insufficient to define CE for infertile women based on ESPC density alone. Several studies, however, point out that the diagnostic criterion of 1 ESPC in 10 HPFs potentially leads to the overdiagnosis of histopathologic CE [41,42]. Intriguingly, McQueen et al. [36] reported that the detection of “endometrial stromal changes” (characterized by the spindling of cells, edema, breakdown, pigment deposition, areas of hypercellularity, and the presence of lymphocytes, eosinophils, neutrophils, and histiocytes), along with CD138(+) ESPCs, could potentially reduce the false-positive rate in the histopathologic diagnosis of CE. The findings suggest that the combined evaluation of the enumeration of CD138(+) ESPCs and careful observation of the other morphological characteristics of endometrial cells may potentially elevate the diagnostic accuracy of histopathologic CE. Further well-designed studies are indispensable in identifying infertile women with CE who would benefit from antibiotic treatment.

## 3. Challenges in Clinical Diagnosis of Hysteroscopic CE: How Do We Reach a Consensus between Histopathologic CE and Hysteroscopic CE?

Fluid hysteroscopy is a handy tool that enables real-time visualization of the uterine cavity and has been widely utilized in gynecologic practice. In 2019, based on a systematic review of past publications and the agreement of the Delphi poll, the International Working Group for the Standardization of Chronic Endometritis Diagnosis proposed the following hysteroscopic diagnostic criteria for CE [43]:

(1) Strawberry aspect: first described by Cravello et al. [44], recognized as large hyperemic localized or scattered mucosal areas flushed with white central points.
(2) Focal hyperemia: small areas of hyperemic mucosa.
(3) Hemorrhagic spots: focal reddish mucosa with sharp and irregular borders, possibly in continuity with capillaries.
(4) Endometrial micropolyps: first described by Cicinelli et al. [45], typically visualized as a cluster of less than 1 mm-sized protrusion on the focal or entire mucosal surface with a distinct connective vascular axis.
(5) Stromal edema: thick and pale appearance of the mucosa in the follicular phase originating from the stromal compartments (a normal finding during the secretory phase).

Among them, endometrial micropolyps are the most unique finding and suggest the presence of histopathologic CE. Using hysteroscopic images and endometrial specimens from 820 women, Cicinelli et al. [45] retrospectively evaluated the relationships between endometrial micropolyps and CE. Endometrial micropolyps were identified in a total of 11.7% of women. The presence of endometrial micropolyps is unexceptionally associated with the presence of other hysteroscopic findings of CE, including focal hyperemia and stromal edema. In 93.7% of women with endometrial micropolyps, histopathologic CE was detected. On the contrary, in women without evident endometrial micropolyps, the prevalence of histopathologic CE was less frequent (10.8%). Thus, the likelihood of histopathologic CE was very high in women with endometrial micropolyps (odds ratio: 124.2, confidence interval: 50.3–205.4). Meanwhile, endometrial micropolyps were detected in 53.6% of women diagnosed with histopathologic CE. The sensitivity, specificity, and positive and negative predictive values of the endometrial micropolyps for histopathologic CE were 54%, 99%, 94%, and 89%, respectively, resulting in a diagnostic accuracy of 90%.

Conversely, in a study investigating the association of hysteroscopic findings with histopathologic CE, Zolghadri et al. [46] reported higher sensitivity (98.4%) and negative predictive values (97.82%), along with lower specificity (56.23%) and positive predictive values (63.5%) of the combination of endometrial micropolyps and hyperemia for histopathologic CE. The limitation of these studies was that the diagnosis of histopathologic CE relied on the findings of superficial stromal edema, increased stromal density, and pleomorphic stromal infiltrates of lymphocytes and plasma cells based on classical tissue staining using hematoxylin-eosin, but not on IHC-CD138.

Using IHC-CD138, we retrospectively assessed the relationship between endometrial micropolyps and histopathologic CE in 52 infertile women with RIF [47]. When histopathologic CE was defined as ≥2.5 CD138(+) ESPC in 10 HPFs, the sensitivity, specificity, and positive and negative predictive values of the endometrial micropolyps for histopathologic CE were 65%, 66%, 60%, and 70%, respectively, resulting in a diagnostic accuracy of 65%. Notably, in contrast to endometrial micropolyps, the prevalence of histopathologic CE was 4% in infertile women with a classical endometrial polyp. The limitation of the study was the small sample size.

Song et al. [48] retrospectively searched for hysteroscopic findings relevant to CE in a larger cohort of 1,189 premenopausal women. When histopathologic CE was defined as ≥1 CD138(+) ESPC in 10 HPFs, the sensitivity, specificity, and positive and negative predictive values of the presence of any one of three hysteroscopy features (endometrial micropolyps, hyperemia, and edema) were 59.3%, 69.7%, 42.1%, and 82.8%, respectively; this resulted in a diagnostic accuracy of 66.9%. On the other hand, the specificity (99%) and positive predictive values (64%) increased, whereas the sensitivity (5%) and negative predictive values 73.3% fell when the presence of multiple hysteroscopic features was adopted, which resulted in an increase in diagnostic accuracy (73.5%). The bias of these three studies was their retrospective designs.

In a prospective study that included 94 women with a history of RIF or recurrent pregnancy loss, Bouet et al. [49] reported that the sensitivity, specificity, and positive and negative predictive values of the presence of endometrial micropolyps and hyperemia were 40%, 80%, 35%, and 83%, respectively, resulting in an accuracy of 71%, when histopathologic CE was defined as ≥5 CD138(+) ESPC in 10 HPFs. Endometrial micropolyps, stromal edema, and hyperemia often coexist within an individual, and these three hysteroscopic features are predominant, as suggested by the findings of a histopathologic CE study [50], which were demonstrated in a recent meta-analysis [51]. These findings indicate that the diagnostic accuracy of endometrial micropolyps (alone or combined with other findings) upon hysteroscopy for the prediction of the presence of histopathologic CE based on CD138(+) ESPCs is 60–70%.

On the other hand, strawberry aspect refers to findings first described by Cravello et al. [44], and is recognized as localized or scattered hyperemic mucosal areas flushed with a white central point. Strawberry aspect is reported to be detectable in 65% of women with histopathologic CE. [49]. In addition, there is a positive correlation (16–54% for sensitivity and 60–94% for specificity) between histopathologic CE and strawberry aspect when they are combined with other hysteroscopic findings such as endometrial micropolyps. Lesions with strawberry aspect are very unique but so mild that they may be missed upon hysteroscopy [45].

Intriguingly, a recent study showed a difference in the diagnostic rate of histopathologic CE though its hysteroscopic findings, as well as in the cure rate upon hysteroscopy. Wang et al. [52] performed both hysteroscopy and IHC-CD138 in the proliferative phase for infertile women before proceeding to their first IVF-ET treatment cycles. Hysteroscopic CE was defined as the presence of focal hyperemia/strawberry aspect, endometrial micropolyps, or strawberry edema findings, whereas histopathologic CE was defined as ≥5 ESPCs in 1 HPF (≥50 ESPCs in 10 HPFs). Serum basal follicle-stimulating hormone and progesterone concentration were significantly higher in the endometrial micropolyps group and stromal edema group than in the focal hyperemia/strawberry aspect group. Conversely, body mass index, serum basal luteinizing hormone, anti-Mullerian hormone, and testosterone concentration were significantly higher in the focal hyperemia/strawberry aspect group than in the endometrial micropolyps group and stromal edema group, indicating a high prevalence of focal hyperemia/strawberry aspect in women with polycystic ovarian syndrome. Finally, histopathologic CE was identified in 10.1% of the focal hyperemia/strawberry aspect group, 63.2% of the endometrial micropolyps group, and 74.0% of the stromal edema group. The prevalence of histopathologic CE was significantly lower in the focal hyperemia/strawberry aspect group than in the other two groups, implying a low positive predictive value of hysteroscopic focal hyperemia/strawberry aspect findings for histopathologic CE.

The authors also assessed the effectiveness of oral antibiotic treatment (doxycycline 200 mg/day, 14 days) against hysteroscopic CE in 273 infertile women following a second hysteroscopy performed 3–5 days after the cessation of the menstrual bleeding in the next cycle [52]. The cure of hysteroscopic CE was confirmed in 207 patients (75.82%). While the cure rate of hysteroscopic CE was 73.6% in the endometrial micropolyps group and 83.2% in the stromal edema group, the focal hyperemia/strawberry aspect group was totally resistant to antibiotic treatment (cure rate: 0%). The authors suggest that the focal hyperemia/strawberry aspect may not directly represent the signs of endometrial infection. The prevalence of secondary infertility was higher in the endometrial micropolyps group and stromal edema group than in the focal hyperemia/strawberry aspect group, whereas that of primary infertility was higher in the focal hyperemia/strawberry aspect group than in the other two groups. These findings indicate that infertile women with endometrial micropolyps and/or stromal edema are more likely to have a history of previous pregnancy, and the presence of the conceptus potentially increases the opportunities for microbial infection in the uterine cavity.

As shown by Song et al. [48], so far, studies agree that the more hysteroscopic findings proposed by the International Working Group for the Standardization of Chronic Endometritis Diagnosis are detected, the higher the diagnostic accuracy for the prediction of histopathologic CE. Tsonis et al. [53] demonstrated that the sensitivity, specificity, positive and negative predictive values, and diagnostic accuracy of the presence of endometrial micropolyps alone for histopathologic CE were 37.11%, 93.54%, 41.55%, 92.32%, and 87.33%, respectively. Meanwhile, the sensitivity, specificity, positive and negative predictive values, and diagnostic accuracy of the presence of endometrial micropolyps, stromal edema, hyperemia, and strawberry aspect for histopathologic CE were 18.18%, 99.75%, 82.35%, 94.95%, and 94.77%, respectively.

In summary, if endometrial micropolyps are detected upon fluid hysteroscopy, the likelihood of histopathologic CE is considerably high. By contrast, a substantial proportion of histopathologic CE does not present endometrial micropolyps. If other hysteroscopic features of CE are accompanied by endometrial micropolyps, the predictive accuracy of the presence of the histopathologic CE rises significantly. Meanwhile, the predictive value of endometrial classical polyps for histopathologic CE remains controversial, and further studies are essential to address this question.

## 4. Challenges in Clinical Diagnosis of Microbial CE: What Is the Relationship between Genital-Tract Dysbiosis and CE?

The Human Microbiome Project was a research initiative launched in 2007 by the National Institutes of Health of the United States. Its first phase (HMP1) focused on the identification and characterization of microbiota at the site in the human body. The project revealed that bacterial cells comprise ~3% of human body weight and are present in similar numbers to human somatic cells. Bacterial communities in the human body play an essential role in our health, whereas their imbalance, malfunction, and maldistribution (dysbiosis) can cause a wide range of diseases [54].

*Lactobacillus* is a genus of Gram-positive, facultative, anaerobic (or microaerophilic), rod-shaped, non-spore-forming bacteria. *Lactobacillus* was traditionally thought to be a predominant bacterial genus in the vaginal cavity of healthy premenopausal women. The results of HMP1 confirmed this and identified four *Lactobacillus* species (*L. crispatus*, *L. gasseri*, *L. iners*, and *L. jensenii*) [55]. The critical role of vaginal *Lactobacillus* is the production of lactic acid, which significantly contributes to the maintenance and homeostasis of the mucosal bacterial microenvironment by dropping the local potential of hydrogen [56]. Other molecules synthesized by local *Lactobacillus*, such as bacteriocins and hydrogen peroxide, also contribute to the impaired proliferation of pathogenic micro-organisms.

Meanwhile, the human uterine cavity has long been believed to be in an aseptic condition until recently, when conventional microscopic and culture-based methods were used to detect bacterial communities. High-throughput techniques based on 16s rRNA gene sequence analysis, however, proved the presence of the microbiota in the uterine cavity. In contrast to the literature agreeing that *Lactobacillus* is predominant in the vaginal microbiota, studies on the uterine cavity/endometrial microbiota are controversial. Many studies have described that endometrial microbiota are also dominated by several *Lactobacillus* species (particularly by *L. crispatus* and *L. iners*) [57].

In 2014, Moreno et al. [58] demonstrated that infertile women with *Lactobacillus*-dominant endometrial microbiota (LDM, Lactobacillus composition of 90% or more) have a favorable reproductive outcome in the following IVF-ET cycles. On the contrary, non-LDM were associated with poor reproductive outcomes including implantation failure and pregnancy loss, indicating the importance of endometrial microbial composition for successful embryo implantation and ongoing pregnancy. Endometrial microbiota in infertile women with CE were also characterized in other studies as a decreased proportion of *Lactobacillu*s, along with an increased proportion of other bacterial genera, including *Anaerococcus*, *Bifidobacterium*, *Dialister*, *Gardnerella*, *Prevotella*, *Ralstonia*, *Phyllobacterium*, *Sphingomonas*, *Lactobacillus*, and *Streptococcus* [58,59,60,61].

In most studies describing the benefit of LDM for a successful pregnancy, endometrial samples were obtained via the trans-vagino-cervical route. On the contrary, several studies that took endometrial samples via the trans-peritoneo-myometrial route (i.e., laparoscopy and laparotomy) showed different results. These studies report that bacterial genera other than *Lactobacillus* species, such as *Acinetobacter*, *Pseudomonas*, *Sphingobium*, *Vagococcus*, *Cloacibacterium*, and *Comamonadaceae*, dominated the uterine cavity (non-LDM condition) [62,63,64,65]. One possible explanation for these interstudy discrepancies is potential contamination of the endometrial microbiota with vaginal microbiota in the process of trans-vagino-cervical endometrial sampling, as the estimated bacterial load in the vaginal cavity is reported to be 100- to 10,000-fold more than that in the uterine cavity [62]. Another possibility is that gynecologic diseases with operative indications (such as uterine fibroids and adenomyosis) may alter the endometrial microbiota from an LDM to a non-LDM condition in the women enrolled in the studies. Given these inconsistencies between the studies, it is premature and hazardous to conclude that an endometrial non-LDM condition equals CE and/or dysbiosis in the uterine cavity.

The device for tissue sampling can be a potential source of contamination of the microbiota. *Burkholderia* is a genus of Proteobacteria that shows resistance to multiple antibiotic agents [66]. Some *Burkholderia* species, including *Burkholderia pseudomallei* and *Burkholderia cepacian*, cause serious lethal infectious diseases, such as melioidosis and severe pneumonia in patients with cystic fibrosis [67,68]. On the other hand, *Burkholderia* species are known to be common environmental contaminants. In 2019, we reported that *Burkholderia* was identified in endometrial fluid microbiota (but not in vaginal-secretion microbiota) in a quarter of infertile women with a history of RIF. On the contrary, *Burkholderia* was not detectable in any infertile women undergoing their first IVF-ET attempt [69]. However, after the year 2020, *Burkholderia* became rarely detectable in any of the endometrial fluid and vaginal-secretion microbiota of infertile women [70,71]. Some studies demonstrated that *Burkholderia* is the most prevalent bacterial species and was detected in the uterine cavities of 48.0% of levonorgestrel intrauterine contraceptive system users [72]. These findings highlight the possibility that *Burkholderia* was brought about by medical devices used for infertile women with a history of RIF.

Unlike the misleading results for endometrial microbiota, those for vaginal microbiota are rather clear and may be helpful for the clinical diagnosis of CE. We found that the detection rate and bacterial abundance of *Lactobacillus* in paired endometrial fluid and vaginal-secretion microbiota were at similar levels between infertile women with and without CE. Meanwhile, three lactic acid bacteria (*Streptococcus*, *Enterococcus*, and *Atopobium*) and *Bifidobacterium* were reduced only in vaginal-secretion microbiota (but not in endometrial secretion) in women with CE [70]. Although the study is preliminary and the cause–effect relationship between these findings and CE remains unsolved, the results may hold promise for the microbial diagnosis of CE, not through somewhat interventive endometrial fluid collection, but through less invasive vaginal-secretion sampling.

Genital-tract microbiota analysis is a promising approach to the identification of bacterial species causing CE and to the antibiogram-based choice of antibiotic agents for affected women. More studies, however, are essential before its application as an alternative clinical diagnostic tool for CE.

## 5. Challenges in Clinical Management of CE

### 5.1. Does CE Develop into Endometriosis?

An increasing number of studies have revealed an association between CE and endometriosis, a disease whereby the mucosal tissue, which is morphologically similar to the eutopic endometrium (the lining of the uterine cavity), grows and develops outside the uterus (ectopic endometrioid tissue), causing menorrhalgia, dyspareunia, and female infertility.

In 2014, Takebayashi et al. [73] searched for CE using archival endometrial specimens obtained from women undergoing hysterectomy due to benign gynecologic pathology (Table 2). They found that women with endometriosis have a higher concomitance rate of CE than those without endometriosis (52.9% vs. 27.0%), regardless of their disease stage. In addition, there were no associations between CE and other popular uterine benign diseases, such as leiomyoma and adenomyosis, along with carcinoma in situ of the uterine cervix. The results were similar when two different thresholds were adopted for ESPC density (10 ESPCs or 60 ESPCs in 10 HPFs). These findings were followed by a series of studies demonstrating that CE was identified in 3–53% of patients with endometriosis [74,75,76]. Many of these reports regarding the relationship between CE and endometriosis enrolled premenopausal women undergoing pelvic surgery (hysterectomy or laparoscopy) and who were diagnosed with endometriosis during the operation. The prevalence of CE in women in the general population with suspected endometriosis may be higher, and thus, awaits further studies.

The eutopic endometrium in women with endometriosis shares several common immunological characteristics with that in CE. For example, one feature of the eutopic endometrium in women with endometriosis is unusual infiltration of CD138(+) ESPCs and CD20(+)/CD5(+)/HLA-DR(+) B cells; these are typical immunocompetent cells observed in CE, but are sparse in the nonpathological endometrium [4]. Some studies suggest that these unusual ESPCs and endometrial B cells potentially play a role in the proliferation and survival of the other endometrial cell components. For example, an endometrium with micropolyps has a proliferative nature and contains a larger number of ESPCs than the nonpathological endometrium [47]. Additionally, in the eutopic endometrium with both endometriosis and CE, the expression level of CXCL13, a pro-inflammatory chemokine involved in the selective extravasation of B cells, is upregulated [27,77]. CXCL13 is induced in human endometrial microvascular endothelial cells by microbial antigens such as lipopolysaccharide [27].

Interleukin (IL)-6 and tumor necrosis factor (TNF)-α are cytokines that are detectable at a higher level in the peritoneal fluid of women with endometriosis than in that without endometriosis [78]. The concentration of IL-6 and TNF-α is markedly higher in the menstrual blood of women with CE compared to those without CE [79]. IL-6 is known as a differentiation factor of mature B cells in various tissues. TNF-α raises estrogen biosynthesis in endometrial glandular cells, which may drive the uterine lining to the proliferative phenotype, which may cause the occurrence of endometrial micropolyps [6].

One of the histomorphological characteristics of CE is a delay in endometrial differentiation during the mid-secretory phase, when blastocysts reach and start to implant in this mucosal tissue. About one-third of endometria with CE exhibit proliferative phenotypes, such as pseudostratification and mitotic nuclei in both glandular and surface epithelial cells [27]. Additionally, the gene expression levels of proliferation-associated molecules (*BCL2*, *BAX*, *MKI67*, *ESR1*, *ESR2*, and *PGR*) are aberrantly upregulated in the secretory-phase endometrium with CE [6], whereas genes potentially associated with embryo receptivity (*IL11*, *CCL4*, *IGF1*, and *CASP8*) and decidualization (*PRL* and *IGFBP1*) are downregulated in this period. These findings indicate that the endometrium with CE is unable to respond adequately to progesterone, an ovarian steroid secreted after ovulation, and transforms its component cells into a receptive phenotype for blastocysts; this indicates that CE and endometriosis have characteristics in common regarding endometrial progesterone resistance [80].

Surgical treatment, such as laparoscopic surgery, has been performed for women with endometriosis, but these operations will not always be beneficial for those desiring pregnancy. For example, the removal of ovarian endometrioid lesions is known to induce a postoperative decrease in serum concentration of the anti-Mullerian hormone, a glycoprotein produced by granulosa cells in the preantral and small antral ovarian follicles; this suggests potential impairment of ovarian reserves due to surgical treatment [81].

If endometriosis has a characteristic of infectious disease, antibiotic treatment can be a conservative therapeutic modality. Some mouse studies suggest the effectiveness of antibiotic treatment against endometriotic lesions. Chadchan et al. [82] tested the effect of 21-day oral water-solubilized combined administration of broad-spectrum antibiotics (0.5 mg/mL vancomycin, 1 mg/mL neomycin, 1 mg/mL metronidazole, and 1 mg/mL ampicillin) on endometriotic lesions. Of these four antibiotic agents, metronidazole significantly inhibited pelvic inflammatory responses (suppression of local macrophage proliferation and production of IL-1β, IL-6, and TNF-α) and reduced the volumes and weights of the endometriotic lesions. Intriguingly, oral administration of feces from mice with endometriosis stimulated the regrowth and inflammation of endometriotic lesions in metronidazole-treated mice, indicating a key role of the gut microbiota in the promotion and progression of endometriosis.

Meanwhile, Lu et al. [83] reported the effectiveness of the vaginal administration of a cocktail of vancomycin, neomycin, metronidazole, and ampicillin (once every 3 days for 21 days) via an absorbable gel sponge in mice with endometriotic lesions. While the disorder of vaginal microbiota exacerbated the endometriotic lesions in mice, vaginal antibiotic treatment was capable of reducing the volume of the lesions via regulation of the nuclear factor-kappa B signaling pathway.

Given that there are definitive treatments to achieve both the eradication of endometriotic lesions and the protection of fecundity in women with endometriosis, these animal studies are enticing in terms of non-surgical treatment for infertile women [82,83], although more studies are needed before application to humans

It is not easy to prove if CE can be a precursor lesion to endometriosis. A long-term longitudinal follow-up of women with CE is required to address this issue. Additionally, few appropriate animal models have the characteristics of both ESPC infiltration and cyclic menstruation to test the cause–effect relationship between CE and endometriosis. However, the resemblance in the inflammatory and endocrinological profiles between these two diseases raises our expectations for future research.

### 5.2. How Do We Deal with Antibiotic Resistance in CE?

Antibiotic resistance is a serious global medical problem in the treatment of infectious diseases. Antibiotic resistance is unexceptionally increasing in the field of obstetrics and gynecology. This includes drug-resistant *Neisseria gonorrhea*, *Candida* species, *Mycoplasma genitalium*, group B *Streptococcus*, and some *Escherichia Coli* isolates (ST131) [84,85,86,87]. Multiple antibiotic agents such as penicillin, cephalosporins, macrolides, fluoroquinolones, and trimethoprim/sulfamethoxazole are ineffective against these pathogens. In the treatment of CE, antibiotic agents such as doxycycline, metronidazole, ciprofloxacin, azithromycin, and moxifloxacin have been effective and prescribed for the treatment of CE [6]. Some studies adopted antibiogram-guided antibiotic treatment strategies [88].

However, multi-drug-resistant CE (MDR-CE) is an emerging issue in its clinical management. For example, in 2021, Xiong et al. [5] reported that in 11.0% of cases, CE was resistant to two courses of combined oral antibiotic treatments (levofloxacin lactate, 400 m/day and metronidazole, 1500 mg/day, for 14 days). In 2008, Cicinelli et al. [89] reported that less than in 20% of cases, CE was unaffected by single-course oral doxycycline treatment, whereas their updated data in 2015 demonstrated that in 24.6% of cases, CE was resistant to multiple courses of antibiotic treatments, suggesting an increase in MDR-CE. Few studies, however, have tracked the prevalence of MDR-CE and its transition over time in large and long-term settings.

Between 2010 and 2020, we retrospectively/prospectively surveyed the prevalence of antibiotic resistance in CE in a series of 3449 infertile women with a history of RIF with three or more failed IVF-ET cycles [90]. Resistance to first-line 14-day oral doxycycline treatment (200 mg/day) was found in 21.2% of CE cases. MDR-CE was defined as resistance to first-line oral doxycycline administration and second-line treatment with a combination of metronidazole (500 mg/day) and ciprofloxacin (400 mg/day) for 14 days. the prevalence of MDR-CE in all CE cases increased 8.27-fold from 1.3% (between April 2010 and March 2015) to 9.6% between April 2015 and March 2020 (odds ratio: 8.27, 95% confidence interval: 2.58–26.43, *p* trend < 0.001). Meanwhile, the prevalence of all CE cases did not show marked changes during the decade (30.2% between April 2010 and March 2015 and 31.7% between April 2015 and March 2020, odds ratio: 1.07, 95% confidence interval: 0.90–1.28, *p* trend > 0.05). In some of these patients, using microbiome analysis, we sought the pathogens that are potentially involved in MDR in CE, but microbial genera/species and/or bacterial communities unique to MDR-CE were not identified in the microbiota of their paired endometrial fluid and vaginal-secretion samples [90].

Moxifloxacin is a new-generation fluoroquinolone agent, with a broader spectrum against the bacterial vaginosis-associated species *Atopobium vaginae* and *Gardnerella vaginalis* compared with older-generation ones, including ciprofloxacin. In addition, moxifloxacin exhibits higher activity against Gram-negative bacteria and anaerobes that are superior to metronidazole. Meanwhile, azithromycin is an acid-stable macrolide that has a broader spectrum of antimicrobial activity than erythromycin and covers urethritis and cervicitis due to *Chlamydia trachomatis* or *Neisseria gonorrhoeae*. In a pilot study comparing the effectiveness of oral moxifloxacin (400 mg/day, 10 days) versus oral azithromycin (500 mg/day, 3 days) as third-line empiric antibiotic treatments against MDR-CE in women with a history of RIF, we found a similar cure rate of histopathologic CE (79.2% vs. 75.0%), as well as LBR, in the immediate subsequent embryo transfer cycle (31.6% vs. 33.3%), and in the cumulative three embryo transfer cycles (57.9% vs. 61.1%) [91]. The results suggest that azithromycin may have a clinical advantage over moxifloxacin as a third-line treatment against MDR-CE due to its shorter administration periods, but larger studies are required to confirm these findings. In addition, the cure of persistent histopathologic MDR-CE and live births in subsequent vitrified–warmed blastocyst transfer cycles were obtained in some women with a history of RIF following 14-day, 1500 mg/day oral administration of lincomycin hydrochloride hydrate, a narrow-spectrum lincosamide agent [91].

Some studies claim that re-examination for histopathologic CE following first-line doxycycline treatment is clinically insignificant in improving reproductive outcomes in infertile women undergoing assisted reproductive technology treatment [92]; however, a growing number of studies demonstrate that antibiotic treatment may improve the reproductive outcomes of infertile women with a history of RIF in subsequent IVF-ET cycles only if the cure of histopathologic CE is confirmed in the endometrial biopsy/IHC-CD138 afterward [20]. Otherwise, no sufficient evidence has been shown for improvements in reproductive outcomes in RIF women with persistent CE.

For infertile women with CE accompanied by uterine cavity deformities, hysteroscopic resection of these lesions may be a potential therapeutic option to reduce the need to prescribe antibiotic agents. Kuroda et al. [93,94], reported a high prevalence of histopathologic CE with ≥5 ESPCs/10 HPFs in infertile women with uterine cavity deformities (85.7% with endometrial polyps, 69.0% with submucosal uterine fibroids, 78.9% with intrauterine adhesions, and 46.2% with septate uterus). A multivariate analysis disclosed that CE was diagnosed more often in women with endometrial polyps (odds ratio: 27.69; 95% confidence interval: 15.01–51.08) and in the intrauterine adhesion groups (odds ratio: 8.85; 95% confidence interval: 3.26–24.05). The cure rate of histopathologic CE following hysteroscopic surgery without antibiotic treatment in women with endometrial polyps, submucosal uterine fibroids, intrauterine adhesions, and septate uterus was 89.7%, 100%, 92.8%, and 83.3%, respectively. Xiang et al. [95] confirmed a high prevalence of histopathologic CE in infertile women with severe intrauterine adhesions than in women with moderate ones (28.6% versus 15.1%). Following combined therapy of hysteroscopic resection of the lesions and single 14-day oral antibiotic treatment, the OPR and MR in these women in subsequent ET cycles were comparable to those in women without CE. Hysteroscopic surgery may be a novel treatment option for CE with endometrial micropolyps or stromal edema, although further studies are necessary to confirm the results.

## 6. Conclusions

A growing body of evidence supports the idea that CE is associated with poor reproductive outcomes in infertile women [96]. CE potentially disturbs the local microenvironment for migrating and embedding embryos in the uterine cavity, which can lead to implantation failure, pregnancy loss, and obstetric complications. Meanwhile, the causality between CE and human reproduction remains undetermined. Researchers, so far, have utilized their own definitions of CE. To provide answers to unsolved questions on CE, the establishment of universal diagnostic criteria that integrate histopathology, hysteroscopy, and microbiome analysis is an urgent issue.

## Figures and Tables

**Table 1:** Predominance of endometrial stromal CD138(−) cells with spoke-wheel/clock-face appearance over endometrial stromal CD138(+) cells with the same appearance in CE. A total of 1568 endometrial stromal cells were evaluated in the archival photographs (×400 magnifications) of the endometrial IHC-CD138/hematoxylin-eosin-stained sections of seven CE cases (*p* < 0.0001 according to Fischer’s exact test).

	Endometrial Stromal Cells with Spoke-Wheel/Clock-Face Appearance	Endometrial Stromal Cells without Spoke-Wheel/Clock-Face Appearance
Endometrial stromal CD138(+) cells	204 (13.0%)	75 (4.8%)
Endometrial stromal CD138(−) cells	427 (27.2%)	862 (55.0%)

**Table 2:** Publications on the relationship between histopathologic CE and endometriosis.

Article/Ethnicity/Study Period/ Study Design/Sample Source	Age (Years)/BMI (kg/m^2^) (Endometriosis Group vs. Control Group)	Stage of Endometriosis (Revised American Society for Reproductive Medicine Classification)	Diagnostic Criteria for CE	Prevalence of Histopathologic CE in Endometriosis Group	Prevalence of Histopathologic CE in Control Group	*p*-Value
Kitaya et al. [31]/Japanese/ January 2002–December 2010/ Retrospective/Hysterectomy specimens	Information unavailable	Information unavailable	5 or more ESPCs in 10 HPFs (400-fold magnification)	5.00% (1/20)	11.68% (25/214) (non-endometriosis, endometrial benign diseases)	0.7072
Takebayashi A et al. [73]/Japanese/ April 2001–December 2012/Retrospective/Hysterectomy specimens	44.15, 3.65 vs. 43.15, 2.75 (mean, SD) (*p* = 0.711)/ 22.08, 4.83 vs. 21.60, 3.14 (mean, SD) (*p* = 0.940)	Stage I-IV (no relationship between the prevalence of CE and stage)	5 or more ESPCs in 10 HPFs (400-fold magnification)	52.94% (18/34)	27.02% (10/37) (non-endometriosis, endometrial benign diseases)	0.0311
Cicinelli E et al. [74]/Italian /January 2010–June 2016/ Retrospective/Hysterectomy specimens	44.3, 2.8 vs. 44.0, 2.3 (mean, SD) (*p* >0.05)/ 27.3, 4.2 vs. 27.2, 4.3 (mean, SD) (*p* >0.05)	Stage IV	1 or more ESPCs in 10 HPFs (100-fold magnification)	38.46% (30/78)	14.10% (11/78) (non-endometriosis, endometrial benign diseases)	<0.001
Freitag N et al. [75]/German (>90% Caucasian)/January 2013–February 2017/Retrospective/Pipelle suction specimens	26-48 (range)/ (Information unavailable on BMI)	Information unavailable	5 or more ESPCs per mm^2^ section	12.90% (8/62)	10.00% (5/50) (non-endometriosis, infertility)	0.634
Khan KN et al. [76]/Japanese/ April 2015–February 2017 Prospective, non-randomized/ Curettage specimens	18-51 vs. 26-51 (range)/ Information unavailable on BMI	Stage I-IV (no relationship between the prevalence of CE and stage)	1 or more ESPCs in 5 HPFs (200-fold magnification)	≥22.6% (≥12/53) not examined prior to treatment/33.4% (7/21)	≥23.4% (≥11/47) not examined prior to treatment/27.3% (3/11)	1.000 *

Footnote. * Calculated by one of the authors (K.K.).

## Data Availability

Not applicable.
